# On the Remedial Properties of Alimentary Substances, and the Changes Produced by Oxygen in Health and Disease, Being an Address Delivered before the Illinois State Medical Society

**Published:** 1851-10

**Authors:** 


*On the Remedial Properties of Alimentary Substances, and the Changes Produced by Oxygen in Health and Disease, being an Address Delivered before the Illinois State Medical Society.* By W. B. HERRICK, M. D., President of the Society; Prof. of Anatomy and Physiology in the Rush Medical College. Published by order of the Society.

The author of this address evinces much ability in the discussion of the subjects selected. His enthusiasm, however, carries him beyond the facts pertaining to the present state of science, into the sea of speculation. He manages his craft adroitly, but, if we do not err, the sounding line will give warning of shoals, or a lee shore. The address, however, is well worthy a perusal. For the lover of *new views* it contains food for reflection. We are constrained to think that the author's ideas are visionary, yet, like many fictions, they are founded on fact. We had designed to review the address at considerable length, but our limited space compels us to content ourselves with this brief notice.

